# Assessment of Cardiovascular Risk Factors in Patients with Juvenile Idiopathic Arthritis

**DOI:** 10.3390/nu15071700

**Published:** 2023-03-30

**Authors:** Marta Gruca, Justyna Zamojska, Katarzyna Niewiadomska-Jarosik, Agnieszka Wosiak, Aleksandra Stasiak, Karolina Sikorska, Jerzy Stańczyk, Elżbieta Smolewska

**Affiliations:** 1 Department of Pediatric Cardiology and Rheumatology, Medical University of Lodz, Sporna 36/50, 91-738 Lodz, Poland; 2 Institute of Information Technology, Lodz University of Technology, 91-738 Lodz, Poland; 3 Paediatric Department, Independent Public Health Care Complex in Minsk Mazowiecki, Szpitalna 37, 05-300 Warsaw, Poland

**Keywords:** juvenile idiopathic arthritis, nutrition, physical activity, prevention, cardiovascular disease, chronic disease

## Abstract

Introduction: The aim of this study was to assess the exposure to cardiovascular disease (CVD) risk factors in patients with juvenile idiopathic arthritis (JIA). Intima–media complex thickness (IMT), selected metabolic parameters and health behaviors were assessed in the course of the study. Methods: The study included study group, which consisted of 45 patients with JIA and 37 healthy age- and sex-matched children in the control group. Analyses in both groups included anthropometric parameters, laboratory tests, IMT and a questionnaire on exposure to modifiable CVD risk factors. Results: The study confirmed that CVD risk factors were present in both groups of patients. Significantly more children with JIA had abnormal BMI (*p* = 0.006) compared to the control group. Children in the study group were more likely to consume fruit regularly (*p* = 0.021) and less likely to consume fast food (*p* = 0.011) and sweetened beverages (*p* = 0.042) than children in the control group. Only 1 patient with JIA met criteria for ideal cardiovascular health. Dietary habits were not associated with IMT values, BMI, presence of joint pain or biochemical parameters in the study group. Conclusions: Patients with JIA are exposed to cardiovascular risk factors equally to their healthy peers. Ideal cardiovascular health should be pursued in the pediatric population with particular attention paid to patients with chronic diseases (i.e., JIA). The application of carotid artery IMT measurement in the assessment of CVD risk requires studies on a larger group of patients.

## 1. Introduction

Juvenile idiopathic arthritis (JIA) represents the most common rheumatic disease in the pediatric population, defined as persistent joint inflammation with a duration of at least 6 weeks, onset before the age of sixteen and of unknown etiology [1]. Certain groups of pediatric patients, including JIA patients, are considered to have a greater risk of developing CVD and their complications than the general population due to the chronic nature of the disease [2]. Despite significant advances in treatment, increased accessibility to the most advanced biological treatment programs and research into personalized therapy, the disease is still associated with an increased risk of developing CVD in adulthood [3]. Cardiovascular complications in adult patients with rheumatoid arthritis (RA) include ischemic heart disease, which is a significant cause of mortality in the RA patient population compared to the general population [4,5]. The increased risk of CVD in adult patients with RA, as well as children and adolescents with JIA, is believed to be affected by chronic inflammation [6]. In as many as 40% of JIA patients, chronic inflammation may persist into adulthood [7]. Considering the higher risk of developing CVD in JIA patients, it is important to seek methods of assessing cardiovascular changes in this group of patients, which will give the opportunity to implement early interventions in this group of patients [8].

Ideal cardiovascular health in the pediatric population is defined as the presence of four health-promoting behaviors (lack of exposure to secondhand smoking, BMI <85th percentile, 60 min of physical activity per day and healthy dietary habits) and three health indicators (correct concentration of total cholesterol, normal blood pressure, normal fasting blood glucose levels). According to the criteria of ideal cardiovascular health established by the AHA in 2016, a healthy diet should be defined as daily consumption of fruits and vegetables, consumption of fish at least twice a week and refraining from carbonated and sweetened beverages [9]. Although the clinical manifestation of CVD and their complications are rare in the pediatric population, children and adolescents are exposed to risk factors that predispose them to the development of CVD and result in higher incidence of CVD in adulthood [10,11]. Other risk factors contributing to the development of CVD include lack of adequate physical activity, with several studies showing that patients with JIA are less active than their healthy peers [12,13]. It is important to remember that both overweight and obesity, as well as underweight, are risk factors for the development of cardiovascular disease both in the general population and among patients with RA [14,15]. This also applies to JIA patients, who may demonstrate abnormally low as well as abnormally high body weight [16]. It is commonly known that weight fluctuations (overweight and obesity) are closely associated with the occurrence of abnormally high blood pressure values and abnormal fasting glucose, which is also relevant to JIA patients [17,18,19,20]. Unhealthy diet in the general population, as well as in JIA patients, is associated with abnormal lipid profile values and development of atherosclerotic plaque [8]. Promoting a healthy diet is important, especially among patients who are initially burdened with a higher risk of developing CVD than the general population [8]. Another important risk factor for the development of CVD in the pediatric population is exposure to passive smoking [21]. Only a few studies has analyzed the prevalence of smoking among adolescents and adults with JIA; however, chronic diseases, defined as disability or illness lasting >6 months and requiring ongoing medical care, are a confirmed risk factor predisposing them to smoking in the teenage years and early adolescence [8,22]. In addition, exposure to tobacco smoke from fetal life to early childhood may be a risk factor for the development of JIA [23,24,25].

The evaluation of the intima–media complex thickness (IMT) of the common carotid arteries is one of the simple, non-invasive methods for assessing cardiovascular changes in children. It is one of the most well validated methods for CVD risk assessment, which predicts the onset of atherogenesis and thus the occurrence of future cardiovascular events in the general population [26]. Meta-analyses available in the literature conclude that RA patients have accelerated atherosclerosis and increased IMT compared to healthy subjects [27,28]. A positive association between active smoking and higher IMT values was proven in the adult population [29]. Studies evaluating the role of carotid IMT in the JIA patient population are inconclusive; therefore, conducting such an analysis on a larger group of JIA patients would be a valuable addition [30,31,32,33].

Despite the fact that JIA is the most common rheumatic childhood disease, children with this disease entity represent a small portion of the pediatric population. Therefore, there is a lack of studies evaluating exposure to CVD risk factors among this group of patients. Based on the studies referenced in the introduction, it would seem that children with JIA are at higher risk of developing CVD than their healthy peers. Our study aims to evaluate diet, physical activity, exposure to secondhand smoking, body weight, IMT complex and selected metabolic parameters in children with JIA. Cardiovascular disease prevention measures must be applied at an early age to be effective; thus, it is useful to identify children at the highest risk of developing CVD and its complications among JIA patients in order to implement preventive measures early on.

## 2. Materials and Methods

### 2.1. Participants

The study included a study group, which consisted of 45 patients with JIA, and 37 healthy age- and sex-matched children, who served as a control group. All children underwent physical examination with assessment of anthropometric parameters, including height and weight. The BMI was calculated based on the anthropometric assessment. The ideal BMI according to the AHA report was considered to be <85th percentile. Underweight was defined as BMI <5th percentile, overweight was defined as BMI between the 85th and 95th percentile, whereas obesity was defined as BMI >95th percentile referenced on centile grids appropriate for age and sex. All patients had three separate oscillometric blood pressure measurements with a sphygmomanometer and an appropriate cuff adapted to the length and circumference of the child’s arm. In addition, the parent of each child completed a questionnaire regarding perinatal history of the child, information about physical activity (all forms of physical activity), time spent in front of device screens (TV, computer or tablet during the day), dietary habits (daily consumption of vegetables and fruits, fish, sweetened beverages, sweets, energy drinks), secondhand smoking and family history of cardiovascular diseases. An abbreviated nutritional questionnaire focused on selected elements of a diet in a week prior to completion of the survey was also executed from each participant and their parents. Respondents were given the opportunity to ask questions and had adequate time to complete the questionnaire, which reduced the possibility of survey bias. The joint pain experienced by JIA patients was assessed using a visual analogue scale (VAS). Blood samples of approximately 3 mL were collected from all patients after 8–12 h of fasting. The serum collected during the course of the study was used to measure concentrations of glucose, total cholesterol, high- and low-density lipoproteins, triglycerides, uric acid, creatinine, alanine transaminase (ALT), erythrocyte sedimentation rate (ESR) and C-reactive protein (CRP), among others. Ultrasonographic cIMT measurements were performed by a single certified examiner with a Toshiba Aplio 400 ultrasonograph with a 12 MHz linear transducer. Both right (RCA) and left (LCA) carotid arteries were assessed and their cIMT values calculated in accordance with percentiles. The procedure was performed according to the recommendations of Pignoli et al. [34], as well as described in other Polish studies [33,35]. The common, internal and external carotid arteries were identified using B-mode and color Doppler ultrasonography. A careful search was performed to obtain an optimal visualization of the vessel wall highlighting the typical double line representing the intima–media layer. Three measurements were taken for each carotid artery, extracting the mean value from the thickness complex. The characteristics of the group are presented in Table 1 and Table 2. There were no missing values in the data gathered and no outliers to handle separately. Questionnaires on exposure to modifiable CVD risk factors were analyzed in both groups. The questions in the questionnaires did not contain sensitive data. The study was approved by the Bioethics Committee of the Medical University of Lodz, Poland (Approval No. RNN/101/19/KB issued on 12 February 2019). Informed, written consent was obtained from each participant (written consent if the patient was above the age of 16 and verbal below the age of 16) and their legal guardian prior to the study.

### 2.2. Statistical Analysis

The continuous variables were presented as median, mean and standard deviation values, and the qualitative variables were presented as proportions. The normality of the distribution was verified by the Kolmogorov–Smirnov test. The statistical significance of differences between the mean values of continuous independent variables with a normal distribution was assessed using Student’s *t*-test, and of continuous independent variables with abnormal distribution using the Mann–Whitney U test. Categorical variables were compared using the chi2 test incorporating Yates’ correction for continuity where indicated. For readability, the presented tables contained global *p*-values if there were more than two levels in parameters. Differences were considered statistically significant when the coefficient of statistical significance was below 0.05 (*p*-value < 0.05).

## 3. Results

Based on the information provided on children’s health-seeking behaviors and the results of additional tests (including laboratory tests and anthropometric measurements), the ideal cardiovascular health status was stated in only one patient with JIA (2.22%) and no patient in the control group. The difference between the groups was not statistically significant (*p* = 0.904).

The ideal BMI (<85th percentile) was obtained in 27 (60.00%) patients with JIA and 29 (78.38%) healthy controls (*p* = 0.075), and an appropriate BMI (values between 5 and 85 percentile; excluding underweight) was determined in 22 (48.89%) JIA and 29 (78.38%) healthy controls; the difference between these groups was statistically significant (*p* = 0.006).

Overweight was determined in 11 (24.44%) children with JIA and 7 (18.92%) children from the control group. Obesity was identified in the case of seven (15.56%) children with JIA and one (2.70%) child from the control group. Underweight was found in only four (8.89%) patients with JIA; there were no correlations stated between these groups. The criterion of adequate physical activity (physical activity >60 min, 7 days per week) was recorded for only eight JIA patients (17.78%) and five (13.59%) healthy peers; the difference between groups was not statistically significant (*p* = 0.599).

Nine children with JIA (20.0%) and five (13.51%) healthy peers in the control group were exposed to tobacco smoke in their immediate environments. The difference between groups was not statistically significant (*p* = 0.437). Relationships between normal and abnormal IMT values and selected parameters were analyzed and presented in Table 3. The differences within the evaluated parameters were not statistically significant—they did not depend on the level of the IMT complex value. This is due to the fact that only four patients in the study group had an abnormal IMT complex value, which emphasizes the necessity to conduct a study on a larger group of patients in order to demonstrate the relationship between the values.

There were statistically significant differences in the frequency of fruit consumption between children in the control and study groups—all children in the study group consumed fruits at least once a week, but in contrast, significantly more children in the control group consumed fruit 5–6 days a week, while children in the study group usually consumed fruit 2–4 days a week (*p* = 0.003 for post hoc analysis of compared pairs). Children in the study group were significantly less likely to drink sweetened beverages and consume fast foods than their healthy peers (*p* = 0.011).

Based on automated K-means clustering analysis, the study group was divided into four diet-dependent clusters (Table 4).

The diet of children in the study group, depending on the cluster to which they belonged to, was not related to physical activity, BMI, lipid profile values or pain complaints. In the automatic dietary breakdown, the most significant differences between groups were those in the consumption of fruits, sweets, fast foods and sweetened beverages. These are the elements of nutrition that should receive the most attention when improving dietary habits of JIA patients. Table 5 shows the relationships between selected parameters and diet. There were also no significant correlations between the dietary regimen and the values of the IMT complex, sedentary screen time or ESR values. This may be due to too small a sample of children in the study group. Due to the fact that according to the AHA criteria only one child with JIA fulfilled the criterion of a healthy diet, an analysis of dietary habits was also conducted, in which good and poor eating habits were distinguished. No child in the control group met the mentioned AHA criteria. Good eating habits included daily consumption of fruits or vegetables, consumption of fish ≥1 time/week and consumption of sweetened beverages less than 1 time/week. Bad eating habits were, respectively, consumption of fruits or vegetables less than daily, consumption of fish <1 × per week and consumption of sweetened beverages more than 1 × per week (Supplementary Materials Table S1—overall study group and controls and Table S2—only study group). There was no correlation between pain complaints and other parameters in the study group (Supplementary Materials Table S3).

## 4. Discussion

Only a few studies available in the literature assessed the cardiovascular health of the pediatric population. However, it should be emphasized that atherosclerotic plaque develops in early childhood [36], which is why prevention programs, which have been proven effective in the adult population [37], are also of such importance in the pediatric population. Young adults with JIA have subclinical atherosclerosis, even if their arthritis is well controlled with or without pharmacological therapy [3]. In our previous study we have shown higher exposure to CVD risk factors among children in professional physician families compared to the general population. Alarming conclusions were drawn that even among a professional group of physicians with knowledge of the harmful exposure to CVD risk factors, only 22 children (5.6% of all subjects) had ideal cardiovascular health [38]. In the course of this study we found that only one child with JIA could be classified as having ideal cardiovascular health; this was a child with no exposure to passive smoking, a BMI <85th percentile, daily physical activity for about 1 h and meeting the criterion of a healthy diet, with normal total cholesterol, normal blood pressure and normal fasting blood glucose. On one hand, it is very disturbing that only one child met the criteria for ideal cardiovascular health; on the other hand, it is a positive observation because it can be assumed that parents pay more attention to healthy lifestyle in patients burdened with chronic diseases. However, the group was too small to draw statistically validated conclusions.

### 4.1. Modifiable CVD Risk Factors

Restrictions on access to various forms of physical activity during the COVID-19 pandemic, increased time spent in the front of screens and lack of control of children’s food intake at home contributed to the development of unhealthy eating habits in children [39,40,41].

#### 4.1.1. Sedentary Screen Time

In the adult population, time spent sitting is an independent risk factor for the development of CVD [42]. Several pandemic studies have pointed out the problem of children spending increased periods of time in front of screens on multimedia devices [43,44,45,46]. Although it was not statistically significant in our study, there was a notable increase in sedentary screen time compared to the pre-pandemic period of time in both the study group and the control group (Table 1). Children with JIA have the same risk of sedentary screen time and the resulting side effects as their healthy peers. It should be mentioned that severe visual impairment is reported in up to 38% of patients with JIA (cataract, posterior synechiae, band keratopathy, ocular hypertension, hypotony, macular edema and optic nerve edema) [47]. Among others a positive antinuclear antibody and a shorter duration between the diagnosis of arthritis and uveitis were significantly associated with the presence of ocular complications. Thus, it is therefore important to limit the time spent in front of screens to avoid exacerbating the risk of developing visual dysfunction [48]. In our study, children with an unhealthy diet were found to spend more time in front of a computer screen than children with a healthy diet. In the JIA group there was no correlation between time spent in front of screens and an unhealthy diet (Supplementary Materials Table S2); however, this may be related to the insufficient size of the study group. Similar findings were shown in a study by Myszkowska-Ryciak et al. where the authors found an association between daily screen time exposure and less healthy eating habits. They proved that spending less than 2 h a day in front of screens significantly reduced the odds quotient for adverse dietary behaviors such as drinking sweetened beverages and eating sweets and fast foods; however, no correlation was found regarding screen time and body weight [49]. It has been postulated that the type of performed activity may be crucial for the assessment of the association between screen time and eating behavior. For example, a higher proportion of television viewing in total screen time may be related to the influence of advertising of foods rich in fat, sucrose and salt, promoting greater consumption of these products [50].

#### 4.1.2. Disorders of Body Weight

A significant number of JIA patients present as overweight or obese, but malnutrition is also a significant problem in this group of patients [16]. Patients who are underweight have an increased risk of ischemic heart disease or hypertension [14]. We found that children with JIA had a tendency toward abnormal body weight (overweight, obese, underweight) more than their healthy peers, and the differences between groups were statistically significant (*p* = 0.06). The results we obtained are in line with other investigations conducted in JIA patients. Results of some studies involving patients with JIA indicate a low BMI in this group of individuals. It has not been possible to establish a clear cause of weight deficiency in children with JIA, as it is likely that the occurrence of this phenomenon is multifactorial [16,51]. According to the NCD Risk Factor Collaboration’s 2021 report, as many as 213 million children worldwide are considered to be overweight, and 124 million are obese [52]. A number of authors describe a higher prevalence of overweight and obesity among JIA patients than in the general population of healthy children [53,54]. On the other hand, several studies have been published that did not establish significant differences in BMI in JIA children compared to the general population [55,56,57]. In our study, there was no association between type of diet and BMI in either the JIA patient group or the control group (Supplementary Materials Tables S1, S2 and S4). Our results are consistent with those of Caetano et al. who also found no significant association between energy intake and BMI in JIA patients [58].

#### 4.1.3. Physical Activity

Beneficial health-promoting effects on proper cardiovascular function include an adequate amount of physical activity, which in adults leads to a 20–30% reduction in total mortality and CVD [59]. Furthermore, there is evidence that physical activity patterns established in childhood continue into adulthood [11]. In our study, both patients with JIA and children in the control group did not meet the criterion of proper amounts of physical activity 100% of the time, and the differences between the groups were not statistically significant. Our results are comparable to the work of Israeli and Italian researchers, who found similar levels of activity in the JIA patient group compared to the control group [60]. In contrast, Heale et al. showed that physical activity levels in children with JIA decreased after diagnosis and declined steadily over the course of the study. It is known that psychosocial factors play a significant role in physical activity. The researchers concluded that the promotion of age-appropriate physical activity and safe sporting activities remains an important part of the management of JIA [12]. A study by Miliatz et al. showed that participation in school sports among children and adolescents with JIA has increased significantly over the past 15 years. This is likely due to the availability of new treatment options, which lead to improved functioning of JIA patients [61].

#### 4.1.4. Passive Smoking

Passive smoking in adults with additional chronic diseases (i.e., diabetes) increases the risk of cardiovascular incidents [62]; furthermore, cigarette smokers have worse dietary habits than the general population [63]. Passive smoking in early childhood was proven to cause chronic cardiovascular changes that become clinically apparent in adulthood [64]. Almost one in five Polish adults admits to smoking in the presence of children [65]. A positive association between active smoking and higher IMT values was proven in the adult population [29]. In our study, there was no statistically significant difference between IMT and exposure to secondhand smoking. The aforementioned relationship should be analyzed on a larger group of patients. Our study showed that as many as 20.00% of patients with JIA and 13.51% of healthy controls were exposed to secondhand smoke (the difference was not statistically significant).

#### 4.1.5. Dietary Habits

According to the American College of Rheumatology, there is no evidence that restrictive diets are beneficial in children with JIA [66]. Unnecessary use of restrictive diets, such as gluten-free and dairy-free, can impair nutritional status, delay implementation of treatment and aggravate patients’ clinical conditions. There is no strong evidence that only a specific diet should be applied in the treatment of JIA, but a healthy, well-balanced and age-appropriate diet is essential to support the health and quality of life of a patient with chronic disease [66]. Antioxidant concentrations, including vitamin C, have been shown to be inversely related to the concentration of C-reactive protein and other markers of inflammation. During an inflammatory reaction, there is an increase in the release of reactive oxygen species in damaged areas, balanced by antioxidants. Therefore, in vitamin C deficiency, the antioxidant effect may be lost, contributing to high levels of inflammatory markers, potentially harming patients with JIA [67]. The consumption of adequate amounts of fruits containing vitamin C, which has anti-inflammatory and antioxidant properties, is extremely important in patients with JIA [68]. In our study, children with JIA were more likely to eat fruit than their healthy peers, and the difference between the groups was statistically significant (Table 1). The above results indicate better dietary habits in terms of fruit consumption among children with JIA than their healthy peers. JIA patients consume sweetened fizzy drinks and fast-food less frequently than their healthy peers (Table 1). Our results are different from those of Brazilian researchers, who showed a low intake of fruit and vegetables and a high intake of lipids, sugar and sweets. More than 30% of JIA patients consumed fried foods, soft drinks, artificial juices and/or coffee [58]. However, the authors mentioned that their results were similar to those found in healthy Brazilian children and adolescents [58]. Based on currently available data, we are unable to conclusively determine the cause of the observed correlations. It can be assumed that the results obtained in our study may indicate a greater knowledge of the impact of eating habits on the development of children with JIA. It also cannot be excluded that the change in dietary habits may have been influenced by the overweight or obesity found in children with JIA. The desire to avoid excess weight associated with the effects of steroid use and to prevent excess weight gain through a well-balanced, healthy diet is an interesting topic. At this point, these are only hypotheses that require additional research. It is important to emphasize that fruits and vegetables are part of a healthy diet that can contribute to the health and development of patients with JIA and the management of the disease [69]. Researchers from Texas revealed in their preliminary study that carbohydrate and sugar intake correlated with pain in adolescents with active disease. This correlation was not observed in teenagers in clinical remission or in the control group. These results may have implications for pain management in teenagers with JIA [70]. In our study, there was no correlation between diet and observed pain complaints (Supplementary Materials Table S3). In contrast to the work of Suppasit et al., our study did not show higher blood pressure values or abnormal fasting glucose among patients with JIA compared to controls [19,20].

### 4.2. Assessment of IMT in Patients with JIA

Several previous studies have shown that lifestyle interventions, when implemented in the adult population, can prevent carotid IMT progression [71,72,73] but others have shown no such effects [74]. Assessing the impact of prevention on the development of CVD in children requires long-term follow-up. Nevertheless, it is worth seeking tools to assess the risk and prognosis of CVD development in the pediatric population, particularly among patients with chronic diseases. Only a few projects have evaluated the usefulness of IMT in children with JIA, and the results of these studies are inconclusive [33,35]. In our study, there was no relationship in the study group between IMT complex values and diet (Table 3, Table 4 and Table 5, Supplementary Materials Tables S1 and S2). Similar conclusions were reached by researchers from the University of Minnesota who also found no relationship between IMT values and the type of ingested food (depending on membership in a particular dietary cluster) [75]. This study was conducted on a group of adults with no history of CVD. There is a lack of such studies in the pediatric population, and even more so among children with chronic diseases. Despite this, an important role of dietary pattern analysis is to conduct further research on nutritional levels [75].

## 5. Conclusions

This study brings new data on risk factors for the development of CVD in patients with JIA. During the course of the study, it was confirmed that both JIA patients and their healthy peers are exposed to risk factors for developing CVD. In addition, we noted that patients with JIA try to promote healthy nutritional habits and strive to be as physically active as their healthy peers. Nevertheless, the overall dietary habits of both children with JIA and healthy children require improvement.

Our study emphasizes the need for evaluation of dietary habits by pediatricians who provide ongoing care for children. This would allow for early modification of nutritional habits among children who require it. Such prevention could help avoid the development of overweight or obesity and the resulting complications. We should strive to meet the AHA’s recommendations for healthy eating and physical activity in all children in the pediatric population.

The presented study requires an extension of the research on a larger group of patients, especially in terms of the correlation between the IMT value and risk factors for development of CVD among patients with JIA. It would be also worthwhile to analyze the cause of better dietary habits in children with JIA than in their healthy peers. This requires additional survey research in this group of patients. It is necessary to look for simple tools that can identify the group of children who require specific lifestyle changes, weight reduction and changes in eating habits at an early stage in order to prevent developmental CVD among children who are particularly susceptible. It would be valuable to consider studies evaluating the effects of beneficial lifestyle changes on IMT or selected metabolic parameters in patients with JIA. Such studies, however, would require multi-center collaboration with long-term evaluation of patients at multiple endpoints.

Effective health promoting projects should be applied in order to reduce CVD risk factors in the pediatric population with a special emphasis on patients with chronic diseases. Ideal cardiovascular health should be achieved among the largest possible portion of the child and adolescent population. We should strive for ideal cardiovascular health in the pediatric population.

## Figures and Tables

**Table 1:** Baseline characteristics of the study group.

		Control Group (*n* = 37)		Study Group (*n* = 45)		*p*
		*n*	%	*n*	%	
Sex	F	23	62.16%	33	73.33%	0.279
	M	14	37.84%	12	26.67%	
RCA centile	<25	12	32.43%	22	48.89%	0.116
	25–74	18	48.65%	16	35.56%	
	75–94	7	18.92%	3	6.67%	
	≥95	0	0.00%	4	8.89%	
RCA centile	<75 cc	30	81.08%	38	84.44%	0.687
	≥75 cc	7	18.92%	7	15.56%	
LCA centile	<25	12	32.43%	18	40.00%	0.246
	25–74	18	48.65%	20	44.44%	
	75–94	7	18.92%	3	6.67%	
	≥95	0	0.00%	4	8.89%	
LCA centile	<75 cc	30	81.08%	38	84.44%	0.687
	≥75 cc	7	18.92%	7	15.56%	
cIMT	<95	37	100.00%	41	91.11%	0.145
	≥95	0	0.00%	4	8.89%	
Apgar score	1	0	0.00%	0	0.00%	0.558
	2	1	2.70%	0	0.00%	
	3	0	0.00%	0	0.00%	
	4	0	0.00%	0	0.00%	
	5	0	0.00%	0	0.00%	
	6	0	0.00%	1	2.22%	
	7	0	0.00%	1	2.22%	
	8	7	18.92%	3	6.67%	
	9	11	29.73%	13	28.89%	
	10	18	48.65%	27	60.00%	
Type of weight after childbirth	AGA	33	89.19%	41	91.11%	0.732
	LGA	2	5.41%	3	6.67%	
	SGA	2	5.41%	1	2.22%	
BMI	Underweight	0	0.00%	4	8.89%	0.062
	Appropriate body mass	29	78.38%	23	51.11%	
	Overweight	7	18.92%	11	24.44%	
	Obesity	1	2.70%	7	15.56%	
**BMI**	**normal**	**29**	**78.38%**	**22**	**48.89%**	**0.006**
	**abnormal**	**8**	**21.62%**	**23**	**51.11%**	
BMI < 85	YES	29	78.38%	27	60.00%	0.075
	NO	8	21.62%	18	40.00%	
RR	<90 percentile	36	97.30%	45	100.00%	0.875
	90–95 percentile	1	2.70%	0	0.00%	
	>95 percentile	0	0.00%	0	0.00%	
Ideal cardiovascular health	YES	0	0.00%	1	2.22%	0.904
	NO	37	100.00%	44	97.78%	
Passive smoking	NO	32	86.49%	36	80.00%	0.437
	YES	5	13.51%	9	20.00%	
Smoking parents	NO	34	91.89%	37	82.22%	0.201
	YES	3	8.11%	8	17.78%	
Family history of cardiovascular disease	NO	19	51.35%	25	55.56%	0.704
	YES	18	48.65%	20	44.44%	
ESR	<10 mm/1 h	-------	-------	27	60.00%	------
	>10 mm/1 h	-------	-------	18	40.00%	
Uric acid	<4 mg/L	19	51.35%	25	55.56%	0.704
	≥4 mg/L	18	48.65%	20	44.44%	
Fasting glucose	<100 mg/L	36	97.30%	42	93.33%	0.407
	≥100 mg/L	1	2.70%	3	6.67%	
HDL	<40 mg/dL	2	5.41%	5	11.11%	0.497
	>45 mg/dL	31	83.78%	33	73.33%	
	40–45 mg/dL	4	10.81%	7	15.56%	
HDL	<40 mg/dL	2	5.41%	5	11.11%	0.358
	>45 mg/dL 40–45 mg/dL	35	94.59%	40	88.89%	
LDL	<110 mg/dL	19	51.35%	27	60.00%	0.695
	110–129 mg/dL	9	24.32%	8	17.78%	
	>130 mg/dL	9	24.32%	10	22.22%	
TC	<170 mg/dL	22	59.46%	28	62.22%	0.528
	170–199 mg/dL	13	35.14%	12	26.67%	
	>200 mg/dL	2	5.41%	5	11.11%	
Triglycerides	0–9 years <75 mg/dL 10–19 years <90 mg/dL	22	59.46%	24	53.33%	0.323
	0–9 years 75–99 mg/dL 10–19 years 90–129 mg/dL	10	27.03%	9	20.00%	
	0–9 years >100 mg/dL 10–19 years >130 mg/dL	5	13.51%	12	26.67%	
Lipid profile	normal	9	24.32%	10	22.22%	0.822
	abnormal	28	75.68%	35	77.78%	
CRP	<5 mg/L	-------	-------	41	91.11%	------
	≥5 mg/L	--------	--------	4	8.89%	
RF	minus	------	------	42	93.33%	------
	plus	------	------	3	6.67%	
Painful conditions	No	------	------	36	80.00%	------
	Yes	-------	-------	9	20.00%	
VAS scale	0	------	------	36	80.00%	-------
	1	------	------	5	11.11%	
	2	-------	-------	4	8.89%	
Physical activity	No	7	18.92%	16	35.56%	0.095
	any kind of	30	81.08%	29	64.44%	
Physical activity	No	22	59.46%	26	57.78%	0.878
	≥3 days/week	15	40.54%	19	42.22%	
Number of days with physical activity >60 min per day	0	7	18.92%	16	35.56%	0.051
	1	7	18.92%	4	8.89%	
	2	8	21.62%	6	13.33%	
	3	3	8.11%	6	13.33%	
	4	3	8.11%	4	8.89%	
	5	3	8.11%	1	2.22%	
	6	1	2.70%	0	0.00%	
	7	5	13.51%	8	17.78%	
Physical activity 7 days a week > 60 min	No	32	86.49%	37	82.22%	0.599
	Yes	5	13.51%	8	17.78%	
Sedentary screen time before COVID-19 pandemic	<3 h	10	27.03%	14	31.11%	0.686
	≥3 h	27	72.97%	31	68.89%	
Sedentary screen time in COVID-19 pandemic	<3 h	-------	0.00%	---------	0.00%	------
	≥3 h	37	100.00%	45	100.00%	
**Fruit consumption**	**never**	**0**	**0.00%**	**0**	**0.00%**	**0.021**
	**less than once a week**	**4**	**10.81%**	**0**	**0.00%**	
	**1 time a week**	**3**	**8.11%**	**3**	**6.67%**	
	**2–4 days a week**	**6**	**16.22%**	**18**	**40.00%**	
	**5–6 days a week**	**8**	**21.62%**	**2**	**4.44%**	
	**every day 1 time**	**11**	**29.73%**	**14**	**31.11%**	
	**more than once every day**	**5**	**13.51%**	**8**	**17.78%**	
Vegetables consumption	never	0	0.00%	0	0.00%	0.246
	less than once a week	2	5.41%	0	0.00%	
	1 time a week	0	0.00%	3	6.67%	
	2–4 days a week	11	29.73%	15	33.33%	
	5–6 days a week	10	27.03%	8	17.78%	
	every day 1 time	7	18.92%	16	35.56%	
	more than once every day	7	18.92%	3	6.67%	
Fish consumption	never	3	8.11%	8	17.78%	0.517
	less than once a week	24	64.86%	21	46.67%	
	1 time a week	8	21.62%	13	28.89%	
	2–4 days a week	1	2.70%	2	4.44%	
	5–6 days a week	1	2.70%	0	0.00%	
	every day 1 time	0	0.00%	1	2.22%	
	more than once every day	0	0.00%	0	0.00%	
Sweets consumption	never	0	0.00%	0	0.00%	0.773
	less than once a week	1	2.70%	3	6.67%	
	1 time a week	7	18.92%	9	20.00%	
	2–4 days a week	16	43.24%	20	44.44%	
	5–6 days a week	4	10.81%	2	4.44%	
	every day 1 time	5	13.51%	7	15.56%	
	more than once every day	4	10.81%	4	8.89%	
**Coca-Cola and other fizzy drinks consumption**	**never**	**2**	**5.41%**	**3**	**6.67%**	**0.042**
	**less than once a week**	**7**	**18.92%**	**21**	**46.67%**	
	**1 time a week**	**8**	**21.62%**	**9**	**20.00%**	
	**2–4 days a week**	**13**	**35.14%**	**9**	**20.00%**	
	**5–6 days a week**	**2**	**5.41%**	**0**	**0.00%**	
	**every day 1 time**	**3**	**8.11%**	**0**	**0.00%**	
	**more than once every day**	**2**	**5.41%**	**3**	**6.67%**	
**Fast-food consumption**	**never**	**0**	**0.00%**	**3**	**6.67%**	**0.011**
	**less than once a week**	**14**	**37.84%**	**31**	**68.89%**	
	**1 time a week**	**12**	**32.43%**	**8**	**17.78%**	
	**2–4 days a week**	**10**	**27.03%**	**2**	**4.44%**	
	**5–6 days a week**	**0**	**0.00%**	**0**	**0.00%**	
	**every day 1 time**	**1**	**2.70%**	**1**	**2.22%**	
	**more than once every day**	**0**	**0.00%**	**0**	**0.00%**	
Energy drinks consumption	never	28	75.68%	36	80.00%	0.544
	less than once a week	5	13.51%	6	13.33%	
	1 time a week	3	8.11%	0	0.00%	
	2–4 days a week	1	2.70%	3	6.67%	
	5–6 days a week	0	0.00%	0	0.00%	
	every day 1 time	0	0.00%	0	0.00%	
	more than once every day	0	0.00%	0	0.00%	

cIMT—carotid intima–media thickness; BMI—body mass index; ESR—erythrocytes sedimentation rate; CRP—C-reactive protein; RCA—right carotid artery; LCA—left carotid artery. 1—Abnormal BMI encompasses patients with one of the following: underweight (<5th percentile); overweight (>85th percentile); obesity (>95th percentile); 2—Abnormal lipid panel encompasses patients with at least one of the following: total cholesterol ≥170 mg/dL; high-density lipoprotein ≤40 mg/dL; low-density lipoprotein ≥110 mg/dL; triglycerides 0–9 years >100 mg/dL, 10–19 years >130 mg/dL; Vas—Visual Analog Scale of pain; AGA—appropriate for gestational age; LGA—large for gestational age; SGA—small for gestational age. Statistically significant data are bolded in the table.

**Table 2:** Characteristics by group (control/study).

	Control Group (*n* = 37)			Study Group (*n* = 45)			*p*
	Median	Mean	SD	Median	Mean	SD	
Age	14	13.57	3.051	14	13.31	3.397	0.723
RCA	0.38	0.370	0.044	0.35	0.364	0.058	0.603
LCA	0.37	0.371	0.043	0.36	0.366	0.057	0.625
Birth weight	3.2	3.188	0.420	3.3	3.391	0.514	0.057
ESR	------	------	------	9	12.778	13.075	------

ESR—erythrocytes sedimentation rate; RCA—right carotid artery; LCA—left carotid artery.

**Table 3:** Relationships between the intima media complex and other parameters in the study group.

		IMT < 95 (*n* = 41)		IMT ≥ 95 (*n* = 4)		*p*
		Number	Percent	Number	Percent	
Apgar score	1	0	0.00%	0	0.00%	0.408
	2	0	0.00%	0	0.00%	
	3	0	0.00%	0	0.00%	
	4	0	0.00%	0	0.00%	
	5	0	0.00%	0	0.00%	
	6	1	2.44%	0	0.00%	
	7	1	2.44%	0	0.00%	
	8	2	4.88%	1	25.00%	
	9	11	26.83%	2	50.00%	
	10	26	63.41%	1	25.00%	
Type of weight after childbirth	AGA	37	90.24%	4	100.00%	0.807
	LGA	3	7.32%	0	0.00%	
	SGA	1	2.44%	0	0.00%	
BMI	Underweight	4	9.76%	0	0.00%	0.945
	Appropriate body mass	21	51.22%	2	50.00%	
	Overweight	10	24.39%	1	25.00%	
	Obesity	6	14.63%	1	25.00%	
BMI	Normal	20	48.78%	2	50.00%	0.963
	Abnormal	21	51.22%	2	50.00%	
BMI < 85	YES	25	60.98%	2	50.00%	0.669
	NO	16	39.02%	2	50.00%	
RR	<90 percentile	41	100.00%	4	100.00%	-------
	90–95 percentile	0	0.00%	0	0.00%	
	>95 percentile	0	0.00%	0	0.00%	
Ideal cardiovascular health	YES	1	2.44%	0	0.00%	0.752
	NO	40	97.56%	4	100.00%	
Passive smoking	NO	34	82.93%	2	50.00%	0.116
	YES	7	17.07%	2	50.00%	
Smoking parents	NO	35	85.37%	2	50.00%	0.077
	YES	6	14.63%	2	50.00%	
Family history of cardiovascular disease	NO	23	56.10%	2	50.00%	0.815
	YES	18	43.90%	2	50.00%	
ESR	<10 mm/1 h	25	60.98%	2	50.00%	0.669
	>10 mm/1 h	16	39.02%	2	50.00%	
Uric acid	<4 mg/L	22	53.66%	3	75.00%	0.412
	≥4 mg/L	19	46.34%	1	25.00%	
Fasting glucose	<100 mg/L	38	92.68%	4	100.00%	0.575
	≥100 mg/L	3	7.32%	0	0.00%	
HDL	<40 mg/dL	5	12.20%	0	0.00%	0.450
	>45 mg/dL	29	70.73%	4	100.00%	
	40–45 mg/dL	7	17.07%	0	0.00%	
HDL	<40 mg/dL	5	12.20%	0	0.00%	0.459
	>45 mg/dL 40–45 mg/dL	36	87.80%	4	100.00%	
LDL	<110 mg/dL	24	58.54%	3	75.00%	0.530
	110–129 mg/dL	7	17.07%	1	25.00%	
	>130 mg/dL	10	24.39%	0	0.00%	
TC	<170 mg/dL	26	63.41%	2	50.00%	0.475
	170–199 mg/dL	10	24.39%	2	50.00%	
	>200 mg/dL	5	12.20%	0	0.00%	
Triglycerides	0–9 years <75 mg/dL 10–19 years <90 mg/dL	22	53.66%	2	50.00%	0.410
	0–9 years 75–99 mg/dL 10–19 years 90–129 mg/dL	9	21.95%	0	0.00%	
	0–9 years >100 mg/dL 10–19 years >130 mg/dL	10	24.39%	2	50.00%	
Lipid profile	normal	9	21.95%	1	25.00%	0.889
	abnormal	32	78.05%	3	75.00%	
CRP	<5 mg/L	37	90.24%	4	100.00%	0.513
	≥5 mg/L	4	9.76%	0	0.00%	
RF	minus	38	92.68%	4	100.00%	0.575
	plus	3	7.32%	0	0.00%	
Painful conditions	No	33	80.49%	3	75.00%	0.793
	Yes	8	19.51%	1	25.00%	
VAS scale	0	33	80.49%	3	75.00%	0.410
	1	5	12.20%	0	0.00%	
	2	3	7.32%	1	25.00%	
Physical activity	No	15	36.59%	1	25.00%	0.644
	any kind of	26	63.41%	3	75.00%	
Physical activity	No	25	60.98%	1	25.00%	0.164
	≥3 days/week	16	39.02%	3	75.00%	
Number of days with physical activity >60 min per day	0	15	36.59%	1	25.00%	0.689
	1	4	9.76%	0	0.00%	
	2	6	14.63%	0	0.00%	
	3	5	12.20%	1	25.00%	
	4	3	7.32%	1	25.00%	
	5	1	2.44%	0	0.00%	
	6	0	0.00%	0	0.00%	
	7	7	17.07%	1	25.00%	
Physical activity 7 days a week > 60 min	No	34	82.93%	3	75.00%	0.692
	Yes	7	17.07%	1	25.00%	
Sedentary screen time before COVID-19 pandemic	<3 h	12	29.27%	2	50.00%	0.393
	≥3 h	29	70.73%	2	50.00%	
Sedentary screen time in COVID-19 pandemic	<3 h	0	0.00%	0	0.00%	-------
	≥3 h	41	100.00%	4	100.00%	
Fruit consumption	never	0	0.00%	0	0.00%	0.207
	less than once a week	0	0.00%	0	0.00%	
	1 time a week	2	4.88%	1	25.00%	
	2–4 days a week	15	36.59%	3	75.00%	
	5–6 days a week	2	4.88%	0	0.00%	
	every day 1 time	14	34.15%	0	0.00%	
	more than once every day	8	19.51%	0	0.00%	
Vegetables consumption	never	0	0.00%	0	0.00%	0.435
	less than once a week	0	0.00%	0	0.00%	
	1 time a week	3	7.32%	0	0.00%	
	2–4 days a week	12	29.27%	3	75.00%	
	5–6 days a week	8	19.51%	0	0.00%	
	every day 1 time	15	36.59%	1	25.00%	
	more than once every day	3	7.32%	0	0.00%	
Fish consumption	never	8	19.51%	0	0.00%	0.527
	less than once a week	19	46.34%	2	50.00%	
	1 time a week	12	29.27%	1	25.00%	
	2–4 days a week	2	4.88%	1	25.00%	
	5–6 days a week	0	0.00%	0	0.00%	
	every day 1 time	1	2.44%	0	0.00%	
	more than once every day	0	0.00%	0	0.00%	
Sweets consumption	never	0	0.00%	0	0.00%	0.711
	less than once a week	3	7.32%	0	0.00%	
	1 time a week	9	21.95%	0	0.00%	
	2–4 days a week	17	41.46%	3	75.00%	
	5–6 days a week	2	4.88%	0	0.00%	
	every day 1 time	6	14.63%	1	25.00%	
	more than once every day	4	9.76%	0	0.00%	
Coca-Cola and other fizzy drinks consumption	never	3	7.32%	0	0.00%	0.686
	less than once a week	18	43.90%	3	75.00%	
	1 time a week	9	21.95%	0	0.00%	
	2–4 days a week	8	19.51%	1	25.00%	
	5–6 days a week	0	0.00%	0	0.00%	
	every day 1 time	0	0.00%	0	0.00%	
	more than once every day	3	7.32%	0	0.00%	
Fast-food consumption	never	2	4.88%	1	25.00%	0.507
	less than once a week	28	68.29%	3	75.00%	
	1 time a week	8	19.51%	0	0.00%	
	2–4 days a week	2	4.88%	0	0.00%	
	5–6 days a week	0	0.00%	0	0.00%	
	every day 1 time	1	2.44%	0	0.00%	
	more than once every day	0	0.00%	0	0.00%	
Energy drinks consumption	never	33	80.49%	3	75.00%	0.686
	less than once a week	5	12.20%	1	25.00%	
	1 time a week	0	0.00%	0	0.00%	
	2–4 days a week	3	7.32%	0	0.00%	
	5–6 days a week	0	0.00%	0	0.00%	
	every day 1 time	0	0.00%	0	0.00%	
	more than once every day	0	0.00%	0	0.00%	

cIMT—carotid intima–media thickness; BMI—body mass index; ESR—erythrocytes sedimentation rate; CRP—C-reactive protein; RCA—right carotid artery; LCA—left carotid artery. 1—Abnormal BMI encompasses patients with one of the following: underweight (<5th percentile); overweight (>85th percentile); obesity (>95th percentile); 2—Abnormal lipid panel encompasses patients with at least one of the following: total cholesterol ≥170 mg/dL; high-density lipoprotein ≤40 mg/dL; low-density lipoprotein ≥110 mg/dL; triglycerides 0–9 years >100 mg/dL, 10–19 years >130 mg/dL; VAS—visual analog scale of pain; AGA—appropriate for gestational age; LGA—large for gestational age; SGA—small for gestational age. Statistically significant data are bolded in the table.

**Table 4:** Number of cases in the study group by diet-related clusters.

		Cluster No. 1 (*n* = 7)		Cluster No. 2 (*n* = 3)		Cluster No. 3 (*n* = 19)		Cluster No. 4 (*n* = 16)		*p*
		Number	Percent	Number	Percent	Number	Percent	Number	Percent	
Sex	F	4	57.14%	2	66.67%	13	68.42%	14	87.50%	0.410
	M	3	42.86%	1	33.33%	6	31.58%	2	12.50%	
RCA centile	<25	6	85.71%	3	100.00%	6	31.58%	8	50.00%	0.131
	25–74	1	14.29%	0	0.00%	8	42.11%	6	37.50%	
	75–94	0	0.00%	0	0.00%	1	5.26%	2	12.50%	
	≥95	0	0.00%	0	0.00%	4	21.05%	0	0.00%	
RCA centile	<75 cc	7	100.00%	3	100.00%	14	73.68%	14	87.50%	0.304
	≥75 cc	0	0.00%	0	0.00%	5	26.32%	2	12.50%	
LCA centile	<25	5	71.43%	2	66.67%	5	26.32%	6	37.50%	0.288
	25–74	2	28.57%	1	33.33%	9	47.37%	8	50.00%	
	75–94	0	0.00%	0	0.00%	1	5.26%	2	12.50%	
	≥95	0	0.00%	0	0.00%	4	21.05%	0	0.00%	
LCA centile	<75 cc	7	100.00%	3	100.00%	14	73.68%	14	87.50%	0.304
	≥75 cc	0	0.00%	0	0.00%	5	26.32%	2	12.50%	
cIMT	<95	7	100.00%	3	100.00%	15	78.95%	16	100.00%	0.111
	≥95	0	0.00%	0	0.00%	4	21.05%	0	0.00%	
Apgar score	1	0	0.00%	0	0.00%	0	0.00%	0	0.00%	0.627
	2	0	0.00%	0	0.00%	0	0.00%	0	0.00%	
	3	0	0.00%	0	0.00%	0	0.00%	0	0.00%	
	4	0	0.00%	0	0.00%	0	0.00%	0	0.00%	
	5	0	0.00%	0	0.00%	0	0.00%	0	0.00%	
	6	0	0.00%	0	0.00%	0	0.00%	1	6.25%	
	7	0	0.00%	0	0.00%	0	0.00%	1	6.25%	
	8	0	0.00%	0	0.00%	2	10.53%	1	6.25%	
	9	1	14.29%	1	33.33%	8	42.11%	3	18.75%	
	10	6	85.71%	2	66.67%	9	47.37%	10	62.50%	
Type of weight after childbirth	AGA	6	85.71%	3	100.00%	17	89.47%	15	93.75%	0.360
	LGA	0	0.00%	0	0.00%	2	10.53%	1	6.25%	
	SGA	1	14.29%	0	0.00%	0	0.00%	0	0.00%	
BMI	Underweight	0	0.00%	0	0.00%	3	15.79%	1	6.25%	0.237
	Appropriate body mass	6	85.71%	2	66.67%	9	47.37%	6	37.50%	
	Overweight	1	14.29%	1	33.33%	2	10.53%	7	43.75%	
	Obesity	0	0.00%	0	0.00%	5	26.32%	2	12.50%	
BMI	normal	6	85.71%	2	66.67%	9	47.37%	5	31.25%	0.103
	abnormal	1	14.29%	1	33.33%	10	52.63%	11	68.75%	
BMI < 85	YES	6	85.71%	2	66.67%	12	63.16%	7	43.75%	0.281
	NO	1	14.29%	1	33.33%	7	36.84%	9	56.25%	
RR	<90 percentile	7	100.00%	3	100.00%	19	100.00%	16	100.00%	------
	90–95 percentile	0	0.00%	0	0.00%	0	0.00%	0	0.00%	
	>95 percentile	0	0.00%	0	0.00%	0	0.00%	0	0.00%	
Ideal cardiovascular health	YES	0	0.00%	0	0.00%	0	0.00%	1	6.25%	0.603
	NO	7	100.00%	3	100.00%	19	100.00%	15	93.75%	
**Passive smoking**	**NO**	**7**	**100.00%**	**0**	**0.00%**	**16**	**84.21%**	**13**	**81.25%**	**0.003**
	**YES**	**0**	**0.00%**	**3**	**100.00%**	**3**	**15.79%**	**3**	**18.75%**	
**Smoking parents**	**NO**	**7**	**100.00%**	**0**	**0.00%**	**15**	**78.95%**	**15**	**93.75%**	**0.001**
	**YES**	**0**	**0.00%**	**3**	**100.00%**	**4**	**21.05%**	**1**	**6.25%**	
Family history of cardiovascular disease	NO	3	42.86%	2	66.67%	12	63.16%	8	50.00%	0.741
	YES	4	57.14%	1	33.33%	7	36.84%	8	50.00%	
ESR	<10 mm/1 h	5	71.43%	2	66.67%	11	57.89%	9	56.25%	0.904
	>10 mm/1 h	2	28.57%	1	33.33%	8	42.11%	7	43.75%	
Uric acid	<4 mg/L	6	85.71%	2	66.67%	10	52.63%	7	43.75%	0.296
	≥4 mg/L	1	14.29%	1	33.33%	9	47.37%	9	56.25%	
Fasting glucose	<100 mg/L	5	71.43%	3	100.00%	18	94.74%	16	100.00%	0.078
	≥100 mg/L	2	28.57%	0	0.00%	1	5.26%	0	0.00%	
HDL	<40 mg/dL	1	14.29%	1	33.33%	1	5.26%	2	12.50%	0.543
	>45 mg/dL	5	71.43%	2	66.67%	13	68.42%	13	81.25%	
	40–45 mg/dL	1	14.29%	0	0.00%	5	26.32%	1	6.25%	
HDL	<40 mg/dL	1	14.29%	1	33.33%	1	5.26%	2	12.50%	0.520
	>45 mg/dL 40–45 mg/dL	6	85.71%	2	66.67%	18	94.74%	14	87.50%	
LDL	<110 mg/dL	2	28.57%	3	100.00%	11	57.89%	11	68.75%	0.189
	110–129 mg/dL	1	14.29%	0	0.00%	5	26.32%	2	12.50%	
	>130 mg/dL	4	57.14%	0	0.00%	3	15.79%	3	18.75%	
TC	<170 mg/dL	2	28.57%	3	100.00%	12	63.16%	11	68.75%	0.099
	170–199 mg/dL	2	28.57%	0	0.00%	6	31.58%	4	25.00%	
	>200 mg/dL	3	42.86%	0	0.00%	1	5.26%	1	6.25%	
Triglycerides	0–9 years <75 mg/dL 10–19 years <90 mg/dL	3	42.86%	2	66.67%	12	63.16%	7	43.75%	0.869
	0–9 years 75–99 mg/dL 10–19 years 90–129 mg/dL	2	28.57%	0	0.00%	3	15.79%	4	25.00%	
	0–9 years >100 mg/dL 10–19 years >130 mg/dL	2	28.57%	1	33.33%	4	21.05%	5	31.25%	
Lipid profile	normal	0	0.00%	1	33.33%	6	31.58%	3	18.75%	0.349
	abnormal	7	100.00%	2	66.67%	13	68.42%	13	81.25%	
CRP	<5 mg/L	6	85.71%	3	100.00%	18	94.74%	14	87.50%	0.775
	≥5 mg/L	1	14.29%	0	0.00%	1	5.26%	2	12.50%	
RF	minus	6	85.71%	2	66.67%	18	94.74%	16	100.00%	0.152
	plus	1	14.29%	1	33.33%	1	5.26%	0	0.00%	
painful conditions	No	5	71.43%	3	100.00%	16	84.21%	13	81.25%	0.737
	Yes	2	28.57%	0	0.00%	3	15.79%	3	18.75%	
VAS scale	0	5	71.43%	3	100.00%	16	84.21%	13	81.25%	0.927
	1	1	14.29%	0	0.00%	1	5.26%	2	12.50%	
	2	1	14.29%	0	0.00%	2	10.53%	1	6.25%	
Physical activity	No	1	14.29%	1	33.33%	8	42.11%	6	37.50%	0.621
	any kind of	6	85.71%	2	66.67%	11	57.89%	10	62.50%	
Physical activity	No	3	42.86%	2	66.67%	12	63.16%	9	56.25%	0.807
	≥3 days/week	4	57.14%	1	33.33%	7	36.84%	7	43.75%	
Number of days with physical activity >60 min per day	0	1	14.29%	1	33.33%	8	42.11%	6	37.50%	0.272
	1	0	0.00%	0	0.00%	1	5.26%	3	18.75%	
	2	2	28.57%	1	33.33%	3	15.79%	0	0.00%	
	3	2	28.57%	0	0.00%	3	15.79%	1	6.25%	
	4	1	14.29%	0	0.00%	1	5.26%	2	12.50%	
	5	1	14.29%	0	0.00%	0	0.00%	0	0.00%	
	6	0	0.00%	0	0.00%	0	0.00%	0	0.00%	
	7	0	0.00%	1	33.33%	3	15.79%	4	25.00%	
Physical activity 7 days a week >60 min	No	7	100.00%	2	66.67%	16	84.21%	12	75.00%	0.452
	Yes	0	0.00%	1	33.33%	3	15.79%	4	25.00%	
Sedentary screen time before COVID-19 pandemic	<3 h	2	28.57%	1	33.33%	4	21.05%	7	43.75%	0.548
	≥3 h	5	71.43%	2	66.67%	15	78.95%	9	56.25%	
Sedentary screen time in COVID pandemic	<3 h	0	0.00%	0	0.00%	0	0.00%	0	0.00%	------
	≥3 h	7	100.00%	3	100.00%	19	100.00%	16	100.00%	
**Fruit consumption**	**never**	**0**	**0.00%**	**0**	**0.00%**	**0**	**0.00%**	**0**	**0.00%**	**<0.001**
	**less than once a week**	**0**	**0.00%**	**0**	**0.00%**	**0**	**0.00%**	**0**	**0.00%**	
	**1 time a week**	**0**	**0.00%**	**0**	**0.00%**	**3**	**15.79%**	**0**	**0.00%**	
	**2–4 days a week**	**1**	**14.29%**	**2**	**66.67%**	**15**	**78.95%**	**0**	**0.00%**	
	**5–6 days a week**	**0**	**0.00%**	**1**	**33.33%**	**1**	**5.26%**	**0**	**0.00%**	
	**every day 1 time**	**5**	**71.43%**	**0**	**0.00%**	**0**	**0.00%**	**9**	**56.25%**	
	**more than once every day**	**1**	**14.29%**	**0**	**0.00%**	**0**	**0.00%**	**7**	**43.75%**	
Vegetable consumption	never	0	0.00%	0	0.00%	0	0.00%	0	0.00%	0.294
	less than once a week	0	0.00%	0	0.00%	0	0.00%	0	0.00%	
	1 time a week	0	0.00%	1	33.33%	2	10.53%	0	0.00%	
	2–4 days a week	2	28.57%	2	66.67%	8	42.11%	3	18.75%	
	5–6 days a week	1	14.29%	0	0.00%	5	26.32%	2	12.50%	
	every day 1 time	3	42.86%	0	0.00%	4	21.05%	9	56.25%	
	more than once every day	1	14.29%	0	0.00%	0	0.00%	2	12.50%	
Fish consumption	never	0	0.00%	2	66.67%	3	15.79%	3	18.75%	0.203
	less than once a week	6	85.71%	1	33.33%	9	47.37%	5	31.25%	
	1 time a week	1	14.29%	0	0.00%	5	26.32%	7	43.75%	
	2–4 days a week	0	0.00%	0	0.00%	2	10.53%	0	0.00%	
	5–6 days a week	0	0.00%	0	0.00%	0	0.00%	0	0.00%	
	every day 1 time	0	0.00%	0	0.00%	0	0.00%	1	6.25%	
	more than once every day	0	0.00%	0	0.00%	0	0.00%	0	0.00%	
**Sweets consumption**	**never**	**0**	**0.00%**	**0**	**0.00%**	**0**	**0.00%**	**0**	**0.00%**	**0.019**
	**less than once a week**	**0**	**0.00%**	**0**	**0.00%**	**1**	**5.26%**	**2**	**12.50%**	
	**1 time a week**	**0**	**0.00%**	**0**	**0.00%**	**4**	**21.05%**	**5**	**31.25%**	
	**2–4 days a week**	**0**	**0.00%**	**2**	**66.67%**	**9**	**47.37%**	**9**	**56.25%**	
	**5–6 days a week**	**1**	**14.29%**	**0**	**0.00%**	**1**	**5.26%**	**0**	**0.00%**	
	**every day 1 time**	**4**	**57.14%**	**0**	**0.00%**	**3**	**15.79%**	**0**	**0.00%**	
	**more than once every day**	**2**	**28.57%**	**1**	**33.33%**	**1**	**5.26%**	**0**	**0.00%**	
**Coca-Cola and other fizzy drinks consumption**	**never**	**0**	**0.00%**	**0**	**0.00%**	**1**	**5.26%**	**2**	**12.50%**	**<0.001**
	**less than once a week**	**3**	**42.86%**	**0**	**0.00%**	**8**	**42.11%**	**10**	**62.50%**	
	**1 time a week**	**1**	**14.29%**	**0**	**0.00%**	**6**	**31.58%**	**2**	**12.50%**	
	**2–4 days a week**	**3**	**42.86%**	**0**	**0.00%**	**4**	**21.05%**	**2**	**12.50%**	
	**5–6 days a week**	**0**	**0.00%**	**0**	**0.00%**	**0**	**0.00%**	**0**	**0.00%**	
	**every day 1 time**	**0**	**0.00%**	**0**	**0.00%**	**0**	**0.00%**	**0**	**0.00%**	
	**more than once every day**	**0**	**0.00%**	**3**	**100.00%**	**0**	**0.00%**	**0**	**0.00%**	
**Fast-food consumption**	**never**	**0**	**0.00%**	**0**	**0.00%**	**2**	**10.53%**	**1**	**6.25%**	**0.004**
	**less than once a week**	**3**	**42.86%**	**1**	**33.33%**	**15**	**78.95%**	**12**	**75.00%**	
	**1 time a week**	**3**	**42.86%**	**0**	**0.00%**	**2**	**10.53%**	**3**	**18.75%**	
	**2–4 days a week**	**1**	**14.29%**	**1**	**33.33%**	**0**	**0.00%**	**0**	**0.00%**	
	**5–6 days a week**	**0**	**0.00%**	**0**	**0.00%**	**0**	**0.00%**	**0**	**0.00%**	
	**every day 1 time**	**0**	**0.00%**	**1**	**33.33%**	**0**	**0.00%**	**0**	**0.00%**	
	**more than once every day**	**0**	**0.00%**	**0**	**0.00%**	**0**	**0.00%**	**0**	**0.00%**	
Energy drinks consumption	never	6	85.71%	2	66.67%	15	78.95%	13	81.25%	0.633
	less than once a week	1	14.29%	0	0.00%	3	15.79%	2	12.50%	
	1 time a week	0	0.00%	0	0.00%	0	0.00%	0	0.00%	
	2–4 days a week	0	0.00%	1	33.33%	1	5.26%	1	6.25%	
	5–6 days a week	0	0.00%	0	0.00%	0	0.00%	0	0.00%	
	every day 1 time	0	0.00%	0	0.00%	0	0.00%	0	0.00%	
	more than once every day	0	0.00%	0	0.00%	0	0.00%	0	0.00%	

cIMT—carotid intima–media thickness; BMI—body mass index; ESR—erythrocytes sedimentation rate; CRP—C-reactive protein; RCA—right carotid artery; LCA—left carotid artery. 1—Abnormal BMI encompasses patients with one of the following: underweight (<5th percentile); overweight (>85th percentile); obesity (>95th percentile); 2—Abnormal lipid panel encompasses patients with at least one of the following: total cholesterol ≥170 mg/dL; high-density lipoprotein ≤40 mg/dL; low-density lipoprotein ≥110 mg/dL; triglycerides 0–9 years >100 mg/dL, 10–19 years >130 mg/dL; Vas—Visual Analog Scale of pain; AGA—appropriate for gestational age; LGA—large for gestational age; SGA—small for gestational age; Cluster 1 consisted of children who frequently consume fruits and vegetables, rarely consume fish, frequently consume sweets, moderately often consume sweetened beverages and consume fast foods. Cluster 2 consisted of children who eat fruits and vegetables moderately often but consume sweetened beverages very often (more than once a day). Cluster 3 was created with children who consume fruits quite often, often consume vegetables, consume fish quite rarely, but also quite rarely consume fast foods and moderately rarely consume sweetened beverages. Cluster 4 was made up of children who often consume fruits and vegetables, consume fish and sweets moderately often and rarely consume sweetened beverages and fast food. Statistically significant data are bolded in the table.

**Table 5:** Relationships between selected parameters and diet-related clusters.

	Cluster No. 1 (*n* = 7)			Cluster No. 2 (*n* = 3)			Cluster No. 3 (*n* = 19)			Cluster No. 4 (*n* = 16)			*p*
	Median	Mean	SD	Median	Mean	SD	Median	Mean	SD	Median	Mean	SD	
Age	12	11.86	3.338	16	16.33	1.528	15	14.26	3.07	12	12.25	3.55	0.087
RCA	0.33	0.337	0.030	0.33	0.35	0.044	0.38	0.390	0.068	0.35	0.349	0.048	0.085
LCA	0.34	0.337	0.024	0.33	0.353	0.040	0.37	0.390	0.066	0.36	0.353	0.051	0.144
Birth weight	3.2	3.049	0.678	3.76	3.647	0.402	3.35	3.504	0.494	3.225	3.359	0.437	0.312
ESR	9	8.714	3.988	5	7.333	5.859	9	13.47	11.78	9.5	14.75	17.5	0.653
Sedentary screen time before COVID-19 pandemic [h]	3.5	3.143	1.029	4	3.333	1.155	4	3.789	1.981	3	2.875	1.103	0.375
Sedentary screen time in COVID-19 pandemic [h]	7	7.143	1.574	9	8.667	0.577	9	8.211	1.686	9	7.438	1.861	0.298

ESR—erythrocytes sedimentation rate; RCA—right carotid artery; LCA—left carotid artery; Cluster 1 consisted of children who frequently consume fruits and vegetables, rarely consume fish, frequently consume sweets, moderately often consume sweetened beverages and consume fast foods. Cluster 2 consisted of children who eat fruits and vegetables moderately often but consume sweetened beverages very often (more than once a day). Cluster 3 was created with children who consume fruits quite often, often consume vegetables, consume fish quite rarely, but also quite rarely consume fast foods and moderately rarely consume sweetened beverages. Cluster 4 was made up of children who often consume fruits and vegetables, consume fish and sweets moderately often and rarely consume sweetened beverages and fast food.

## Data Availability

The data used to support the findings of this study are included within the article.

## Supplementary Materials

The following supporting information can be downloaded at: https://www.mdpi.com/article/10.3390/nu15071700/s1, Table S1: Numbers of cases by good and bad eating habits (without division into control and study groups); Table S2: Numbers of cases in the study group divided according to good and bad eating habits; Table S3: Relationships between pain and other parameters in the study group; Table S4: Numbers of cases in the control group divided according to good and bad eating habits.
