# Supplementary material for: Assessment of Cardiovascular Risk Factors in Patients with Juvenile Idiopathic Arthritis
Source: Nutrients. 2023 Mar 30;15(7):1700. doi: 10.3390/nu15071700 (PMC10096608; doi:10.3390/nu15071700)
Supplement: Supplementary file 1 [file nutrients-15-01700-s001.zip › nutrients-2296900-supplementary.pdf]

Table S1. Numbers of cases by good* and bad eating habits (without division into control and study groups)

* fruit or vegetables daily, fish >= 1 time/week, sweetened drinks less than 1 time/week

		Better eating habits (N=10)		Worse eating habits (N=72)		p
		Number	Percent	Number	Percent	
Group	Control	3	30,00%	34	47,22%	0.492
	Study	7	70,00%	38	52,78%	
Sex	F	8	80,00%	48	66,67%	0.237
	M	2	20,00%	24	33,33%	
RCA centile	< 25	5	50,00%	29	40,28%	0.504
	25 – 74	5	50,00%	29	40,28%	
	75 – 94	0	0,00%	10	13,89%	
	>= 95	0	0,00%	4	5,56%	
RCA centile	< 75 cc	10	100,00%	58	80,56%	0.278
	>= 75 cc	0	0,00%	14	19,44%	
LCA centile	< 25	5	50,00%	25	34,72%	0.468
	25 – 74	5	50,00%	33	45,83%	
	75 – 94	0	0,00%	10	13,89%	
	>= 95	0	0,00%	4	5,56%	
LCA centile	< 75 cc	10	100,00%	58	80,56%	0.279
	>= 75 cc	0	0,00%	14	19,44%	
cIMT	<95	10	100,00%	68	94,44%	1.000
	>=95	0	0,00%	4	5,56%	
Apgar score	1	0	0,00%	0	0,00%	0.491
	2	0	0,00%	1	1,39%	

	3	0	0,00%	0	0,00%	
	4	0	0,00%	0	0,00%	
	5	0	0,00%	0	0,00%	
	6	1	10,00%	0	0,00%	
	7	0	0,00%	1	1,39%	
	8	2	20,00%	8	11,11%	
	9	2	20,00%	22	30,56%	
	10	5	50,00%	40	55,56%	
Type of weight after childbirth	AGA	9	90,00%	65	90,28%	0.378
	LGA	0	0,00%	5	6,94%	
	SGA	1	10,00%	2	2,78%	
BMI	Underweight	0	0,00%	4	5,56%	0.486
	Appropriate body mass	5	50,00%	47	65,28%	
	Overweight	3	30,00%	15	20,83%	
	Obesity	2	20,00%	6	8,33%	
BMI	normal	4	40,00%	47	65,28%	0.231
	abnormal	6	60,00%	25	34,72%	
BMI < 85	YES	5	50,00%	51	70,83%	0.335
	NO	5	50,00%	21	29,17%	
RR	<90 percentil	10	100,00%	71	98,61%	0.932
	90-95 percentil	0	0,00%	1	1,39%	
	>95 percentil	0	0,00%	0	0,00%	
Ideal cardiovascular health	YES	1	10,00%	0	0,00%	0.245
	NO	9	90,00%	72	100,00%	
Passive smoking	NO	8	80,00%	60	83,33%	0.852

	YES	2	20,00%	12	16,67%	
Smoking parents	NO	9	90,00%	62	86,11%	0.874
	YES	1	10,00%	10	13,89%	
Family history of cardiovascular disease	NO	8	80,00%	36	50,00%	0.149
	YES	2	20,00%	36	50,00%	
ESR	<10mm/1h	3	30,00%	24	33,33%	0.557
	>10 mm/1h	4	40,00%	14	19,44%	
Uric acid	<4mg/L	6	60,00%	38	52,78%	0.929
	>=4mg/L	4	40,00%	34	47,22%	
Fasting glucose	<100 mg/L	10	100,00%	68	94,44%	1.000
	>=100mg/L	0	0,00%	4	5,56%	
HDL	<40 mg/dl	0	0,00%	7	9,72%	0.528
	>45 mg/dl	9	90,00%	55	76,39%	
	40-45 mg/dl	1	10,00%	10	13,89%	
HDL	<40mg/dl	0	0,00%	7	9,72%	0.670
	>45 mg/dl<br>40-45 mg/dl	10	100,00%	65	90,28%	
LDL	<110 mg/dl	4	40,00%	42	58,33%	0.383
	110-129mg/dl	2	20,00%	15	20,83%	
	>130 mg/dl	4	40,00%	15	20,83%	
TC	<170 mg/dl	5	50,00%	45	62,50%	0.856
	170-199 mg/dl	4	40,00%	21	29,17%	
	>200 mg/dl	1	10,00%	6	8,33%	
Triglycerides	0-9 years <75 mg/dl<br>10-19 years <90 mg/dl	3	30,00%	43	59,72%	0.197

	0-9 years 75-99 mg/dl<br>10-19 years 90-129 mg/dl	4	40,00%	15	20,83%	
	0-9 years >100 mg/dl<br>10-19 years >130 mg/dl	3	30,00%	14	19,44%	
Lipid profile	normal	1	10,00%	18	25,00%	0.513
	abnormal	9	90,00%	54	75,00%	
CRP	<5mg/L	7	70,00%	34	47,22%	0.860
	>=5mg/L	0	0,00%	4	5,56%	
RF	minus	7	70,00%	35	48,61%	0.956
	plus	0	0,00%	3	4,17%	
painful conditions	No	6	60,00%	30	41,67%	0.916
	Yes	1	10,00%	8	11,11%	
VAS scale	0	7	70,00%	30	41,67%	0.408
	1	0	0,00%	5	6,94%	
	2	0	0,00%	3	4,17%	
physical activity	No	4	40,00%	19	26,39%	0.625
	any kind of	6	60,00%	53	73,61%	
physical activity	No	5	50,00%	43	59,72%	0.808
	>= 3 days/week	5	50,00%	29	40,28%	
number of days with physical activity >60 min per day	0	4	40,00%	19	26,39%	0.077
	1	1	10,00%	10	13,89%	
	2	0	0,00%	14	19,44%	
	3	1	10,00%	8	11,11%	
	4	0	0,00%	7	9,72%	
	5	0	0,00%	4	5,56%	

	6	1	10,00%	0	0,00%	
	7	3	30,00%	10	13,89%	
Physical activity 7 days a week > 60 min	No	7	70,00%	62	86,11%	0.398
	Yes	3	30,00%	10	13,89%	
Sedetary screen time before Covid pandemy	< 3h	6	60,00%	18	25,00%	0.049
	>= 3h	4	40,00%	54	75,00%	
Sedetary screen time in Covid pandemy	< 3h	0	0,00%	0	0,00%	------
	>= 3h	10	100,00%	72	100,00%	

cIMT—carotid intima-media thickness; BMI—body mass index; ESR—erythrocytes sedimentation rate; CRP—C-reactive protein; RCA—right carotid artery; LCA—left carotid artery. 1—Abnormal BMI encompasses patients with one of the following: underweight (<5th percentile); overweight (>85th percentile); obesity (>95th percentile); 2—Abnormal lipid panel encompasses patients with at least one of the following: total cholesterol ≥ 170 mg/dL; high-density lipoprotein ≤40 mg/dL; low-density lipoprotein ≥ 110 mg/dL; triglycerides 0-9 years >100 mg/dl, 10-19 years >130 mg/dl; Vas- Visual Analog Scale of pain; AGA- appropriate for gestational age; LGA –large for gestational age; SGA-small for gestational age;

Table S2. Numbers of cases in the study group divided according to good* and bad eating habits

** fruit or vegetables daily, fish >= 1 time/week, sweetened drinks less than 1 time/week*

		Better eating habits (N=7)		Worse eating habits (N=38)		p
		Number	Percent	Number	Percent	
Sex	F	7	100,00%	26	68,42%	0.204

	M	0	0,00%	12	31,58%	
RCA centile	< 25	5	57,14%	18	47,37%	0.616
	25 – 74	3	42,86%	13	34,21%	
	75 – 94	0	0,00%	3	7,89%	
	>= 95	0	0,00%	4	10,53%	
RCA centile	< 75 cc	7	100,00%	31	81,58%	0.504
	>= 75 cc	0	0,00%	7	18,42%	
LCA centile	< 25	4	57,14%	14	36,84%	0.593
	25 – 74	3	42,86%	17	44,74%	
	75 – 94	0	0,00%	3	7,89%	
	>= 95	0	0,00%	4	10,53%	
LCA centile	< 75 cc	7	100,00%	31	81,58%	0.504
	>= 75 cc	0	0,00%	7	18,42%	
cIMT	<95	7	100,00%	34	89,47%	0.860
	>=95	0	0,00%	4	10,53%	
Apgar score	1	0	0,00%	0	0,00%	0.641
	2	0	0,00%	0	0,00%	
	3	0	0,00%	0	0,00%	
	4	0	0,00%	0	0,00%	
	5	0	0,00%	0	0,00%	
	6	1	14,29%	0	0,00%	
	7	0	0,00%	1	2,63%	
	8	1	14,29%	2	5,26%	
	9	1	14,29%	12	31,58%	
	10	4	57,14%	23	60,53%	
Type of weight	AGA	7	100,00%	34	89,47%	0.667

after childbirth	LGA	0	0,00%	3	7,89%	
	SGA	0	0,00%	1	2,63%	
BMI	Underweight	0	0,00%	4	10,53%	0.613
	Appropriate body mass	3	42,86%	20	52,63%	
	Overweight	2	28,57%	9	23,68%	
	Obesity	2	28,57%	5	13,16%	
BMI	normal	2	28,57%	20	52,63%	0.448
	abnormal	5	71,43%	18	47,37%	
BMI < 85	YES	2	28,57%	24	63,16%	0.198
	NO	5	71,43%	14	36,84%	
RR	<90 percentil	7	100,00%	38	100,00%	------
	90-95 percentil	0	0,00%	0	0,00%	
	>95 percentil	0	0,00%	0	0,00%	
Ideal cardiovascular health	YES	1	14,29%	0	0,00%	0.336
	NO	6	85,71%	38	100,00%	
Passive smoking	NO	6	85,71%	30	78,95%	0.916
	YES	1	14,29%	8	21,05%	
Smoking parents	NO	6	85,71%	31	81,58%	0.783
	YES	1	14,29%	7	18,42%	
Family history of cardiovascular disease	NO	6	85,71%	19	50,00%	0.182
	YES	1	14,29%	19	50,00%	
ESR	<10mm/1h	3	42,86%	24	63,16%	0.557
	>10 mm/1h	4	57,14%	14	36,84%	
Uric acid	<4mg/L	4	57,14%	21	55,26%	0.747

	>=4mg/L	3	42,86%	17	44,74%	
Fasting glucose	<100 mg/L	7	100,00%	35	92,11%	0.956
	>=100mg/L	0	0,00%	3	7,89%	
HDL	<40 mg/dl	0	0,00%	5	13,16%	0.576
	>45 mg/dl	6	85,71%	27	71,05%	
	40-45 mg/dl	1	14,29%	6	15,79%	
HDL	<40mg/dl	0	0,00%	5	13,16%	0.716
	>45 mg/dl<br>40-45 mg/dl	7	100,00%	33	86,84%	
LDL	<110 mg/dl	4	57,14%	23	60,53%	0.896
	110-129mg/dl	1	14,29%	7	18,42%	
	>130 mg/dl	2	28,57%	8	21,05%	
TC	<170 mg/dl	4	57,14%	24	63,16%	0.941
	170-199 mg/dl	2	28,57%	10	26,32%	
	>200 mg/dl	1	14,29%	4	10,53%	
Triglycerides	0-9 years <75 mg/dl<br>10-19 years <90 mg/dl	2	28,57%	22	57,89%	0.355
	0-9 years 75-99 mg/dl<br>10-19 years 90-129 mg/dl	2	28,57%	7	18,42%	
	0-9 years >100 mg/dl<br>10-19 years >130 mg/dl	3	42,86%	9	23,68%	
Lipid profile	normal	1	14,29%	9	23,68%	0.956
	abnormal	6	85,71%	29	76,32%	
CRP	<5mg/L	7	100,00%	34	89,47%	0.860
	>=5mg/L	0	0,00%	4	10,53%	
RF	minus	7	100,00%	35	92,11%	0.956

	plus	0	0,00%	3	7,89%	
painful conditions	No	6	85,71%	30	78,95%	0.916
	Yes	1	14,29%	8	21,05%	
VAS scale	0	6	85,71%	30	78,95%	0.541
	1	0	0,00%	5	13,16%	
	2	1	14,29%	3	7,89%	
physical activity	No	4	57,14%	12	31,58%	0.385
	any kind of	3	42,86%	26	68,42%	
physical activity	No	5	71,43%	21	55,26%	0.704
	>= 3 days/week	2	28,57%	17	44,74%	
number of days with physical activity >60 min per day	0	4	57,14%	12	31,58%	0.656
	1	1	14,29%	3	7,89%	
	2	0	0,00%	6	15,79%	
	3	0	0,00%	6	15,79%	
	4	0	0,00%	4	10,53%	
	5	0	0,00%	1	2,63%	
	6	0	0,00%	0	0,00%	
	7	2	28,57%	6	15,79%	
Physical activity 7 days a week > 60 min	No	5	71,43%	32	84,21%	0.783
	Yes	2	28,57%	6	15,79%	
Sedetary screen time before Covid pandemy	< 3h	4	57,14%	10	26,32%	0.240
	>= 3h	3	42,86%	28	73,68%	
Sedetary screen	< 3h	0	0,00%	0	0,00%	------

time in Covid pandemy	>= 3h	7	100,00%	38	100,00%	

cIMT—carotid intima-media thickness; BMI—body mass index; ESR—erythrocytes sedimentation rate; CRP—C-reactive protein; RCA—right carotid artery; LCA—left carotid artery. 1—Abnormal BMI encompasses patients with one of the following: underweight (<5th percentile); overweight (>85th percentile); obesity (>95th percentile); 2—Abnormal lipid panel encompasses patients with at least one of the following: total cholesterol ≥ 170 mg/dL; high-density lipoprotein ≤40 mg/dL; low-density lipoprotein ≥ 110 mg/dL; triglycerides 0-9 years >100 mg/dl, 10-19 years >130 mg/dl; Vas- Visual Analog Scale of pain; AGA- appropriate for gestational age; LGA –large for gestational age; SGA-small for gestational age;

Table S3. Relationships between pain and other parameters in the study group

		Pain = YES (N=9)		Pain = NO (N=36)		p
		Number	Percent	Number	Percent	
Sex	F	7	77,78%	26	72,22%	0.933
	M	2	22,22%	10	27,78%	
RCA centile	< 25	5	55,56%	17	47,22%	0.786
	25 – 74	2	22,22%	14	38,89%	
	75 – 94	1	11,11%	2	5,56%	
	>= 95	1	11,11%	3	8,33%	
RCA centile	< 75 cc	7	77,78%	31	86,11%	0.916
	>= 75 cc	2	22,22%	5	13,89%	
LCA centile	< 25	2	22,22%	16	44,44%	0.660
	25 – 74	5	55,56%	15	41,67%	
	75 – 94	1	11,11%	2	5,56%	
	>= 95	1	11,11%	3	8,33%	
LCA centile	< 75 cc	7	77,78%	31	86,11%	0.916

	>= 75 cc	2	22,22%	5	13,89%	
cIMT	<95	8	88,89%	33	91,67%	0.694
	>=95	1	11,11%	3	8,33%	
Apgar score	1	0	0,00%	0	0,00%	0.987
	2	0	0,00%	0	0,00%	
	3	0	0,00%	0	0,00%	
	4	0	0,00%	0	0,00%	
	5	0	0,00%	0	0,00%	
	6	0	0,00%	1	2,78%	
	7	0	0,00%	1	2,78%	
	8	0	0,00%	3	8,33%	
	9	4	44,44%	9	25,00%	
	10	5	55,56%	22	61,11%	
Type of weight after childbirth	AGA	9	100,00%	33	91,67%	0.669
	LGA	0	0,00%	3	8,33%	
	SGA	0	0,00%	0	0,00%	
BMI	Underweight	1	11,11%	3	8,33%	0.695
	Appropriate body mass	3	33,33%	20	55,56%	
	Overweight	3	33,33%	8	22,22%	
	Obesity	2	22,22%	5	13,89%	
BMI	normal	3	33,33%	19	52,78%	0.502
	abnormal	6	66,67%	17	47,22%	
BMI < 85	YES	4	44,44%	23	63,89%	0.493
	NO	5	55,56%	13	36,11%	
RR	<90 percentil	9	100,00%	36	100,00%	-------
	90-95 percentil	0	0,00%	0	0,00%	

	>95 percentil	0	0,00%	0	0,00%	
Ideal cardiovascular health	YES	0	0,00%	1	2,78%	0.613
	NO	9	100,00%	35	97,22%	
Passive smoking	NO	7	77,78%	29	80,56%	0.780
	YES	2	22,22%	7	19,44%	
Smoking parents	NO	7	77,78%	30	83,33%	0.920
	YES	2	22,22%	6	16,67%	
Family history of cardiovascular disease	NO	3	33,33%	22	61,11%	0.261
	YES	6	66,67%	14	38,89%	
ESR	<10mm/1h	5	55,56%	22	61,11%	0.938
	>10 mm/1h	4	44,44%	14	38,89%	
Uric acid	<4mg/L	4	44,44%	21	58,33%	0.707
	>=4mg/L	5	55,56%	15	41,67%	
Fasting glucose	<100 mg/L	8	88,89%	34	94,44%	0.882
	>=100mg/L	1	11,11%	2	5,56%	
HDL	<40 mg/dl	1	11,11%	4	11,11%	0.917
	>45 mg/dl	7	77,78%	26	72,22%	
	40-45 mg/dl	1	11,11%	6	16,67%	
HDL	<40mg/dl	1	11,11%	4	11,11%	>0.999
	>45 mg/dl 40-45 mg/dl	8	88,89%	32	88,89%	
LDL	<110 mg/dl	5	55,56%	22	61,11%	0.334
	110-129mg/dl	3	33,33%	5	13,89%	

	>130 mg/dl	1	11,11%	9	25,00%	
TC	<170 mg/dl	5	55,56%	23	63,89%	0.875
	170-199 mg/dl	3	33,33%	9	25,00%	
	>200 mg/dl	1	11,11%	4	11,11%	
Triglycerides	0-9 years <75 mg/dl<br>10-19 years <90 mg/dl	6	66,67%	18	50,00%	0.520
	0-9 years 75-99 mg/dl<br>10-19 years 90-129 mg/dl	0	0,00%	9	25,00%	
	0-9 years >100 mg/dl<br>10-19 years >130 mg/dl	3	33,33%	9	25,00%	
Lipid profile	normal	2	22,22%	8	22,22%	0.654
	abnormal	7	77,78%	28	77,78%	
CRP	<5mg/L	9	100,00%	32	88,89%	0.694
	>=5mg/L	0	0,00%	4	11,11%	
RF	minus	9	100,00%	33	91,67%	0.882
	plus	0	0,00%	3	8,33%	
physical activity	No	2	22,22%	14	38,89%	0.586
	any kind of	7	77,78%	22	61,11%	
physical activity	No	5	55,56%	21	58,33%	0.821
	>= 3 days/week	4	44,44%	15	41,67%	
number of days with physical activity >60 min per day	0	2	22,22%	14	38,89%	0.853
	1	2	22,22%	2	5,56%	
	2	1	11,11%	5	13,89%	
	3	1	11,11%	5	13,89%	
	4	1	11,11%	3	8,33%	
	5	0	0,00%	1	2,78%	

	6	0	0,00%	0	0,00%	
	7	2	22,22%	6	16,67%	
Physical activity 7 days a week > 60 min	No	7	77,78%	30	83,33%	0.920
	Yes	2	22,22%	6	16,67%	
Sedetary screen time before Covid pandemy	< 3h	2	22,22%	12	33,33%	0.810
	>= 3h	7	77,78%	24	66,67%	
Sedetary screen time in Covid pandemy	< 3h	0	0,00%	0	0,00%	------
	>= 3h	9	100,00%	36	100,00%	
Better eating habits*	No	8	88,89%	30	83,33%	0.916
	Yes	1	11,11%	6	16,67%	

** fruit or vegetables daily, fish >= 1 time/week, sweetened drinks less than 1 time/week*

cIMT—carotid intima-media thickness; BMI—body mass index; ESR—erythrocytes sedimentation rate; CRP—C-reactive protein; RCA—right carotid artery; LCA—left carotid artery. 1—Abnormal BMI encompasses patients with one of the following: underweight (<5th percentile); overweight (>85th percentile); obesity (>95th percentile); 2—Abnormal lipid panel encompasses patients with at least one of the following: total cholesterol ≥ 170 mg/dL; high-density lipoprotein ≤40 mg/dL; low-density lipoprotein ≥ 110 mg/dL; triglycerides 0-9 years >100 mg/dl, 10-19 years >130 mg/dl; Vas- Visual Analog Scale of pain; AGA- appropriate for gestational age; LGA –large for gestational age; SGA-small for gestational age;

Table S4. Numbers of cases in the control group divided according to good* and bad eating habits

** fruit or vegetables daily, fish >= 1 time/week, sweetened drinks less than 1 time/week*

	Better eating habits (N=3)	Worse eating habits (N=34)	p

		Number	Percent	Number	Percent	
Sex	F	1	33,33%	22	64,71%	0.651
	M	2	66,67%	12	35,29%	
RCA centile	< 25	1	33,33%	11	32,35%	0.841
	25 – 74	2	66,67%	16	47,06%	
	75 – 94	0	0,00%	7	20,59%	
	>= 95	0	0,00%	0	0,00%	
RCA centile	< 75 cc	3	100,00%	27	79,41%	0.916
	>= 75 cc	0	0,00%	7	20,59%	
LCA centile	< 25	1	33,33%	11	32,35%	0.841
	25 – 74	2	66,67%	16	47,06%	
	75 – 94	0	0,00%	7	20,59%	
	>= 95	0	0,00%	0	0,00%	
LCA centile	< 75 cc	3	100,00%	27	79,41%	0.916
	>= 75 cc	0	0,00%	7	20,59%	
cIMT	<95	3	100,00%	34	100,00%	---------
	>=95	0	0,00%	0	0,00%	
Apgar score	1	0	0,00%	0	0,00%	0.999
	2	0	0,00%	1	2,94%	
	3	0	0,00%	0	0,00%	
	4	0	0,00%	0	0,00%	
	5	0	0,00%	0	0,00%	
	6	0	0,00%	0	0,00%	
	7	0	0,00%	0	0,00%	
	8	1	33,33%	6	17,65%	
	9	1	33,33%	10	29,41%	

	10	1	33,33%	17	50,00%	
Type of weight after childbirth	AGA	2	66,67%	31	91,18%	0.079
	LGA	0	0,00%	2	5,88%	
	SGA	1	33,33%	1	2,94%	
BMI	Underweight	0	0,00%	0	0,00%	0.918
	Appropriate body mass	2	66,67%	27	79,41%	
	Overweight	1	33,33%	6	17,65%	
	Obesity	0	0,00%	1	2,94%	
BMI	normal	2	66,67%	27	79,41%	0.828
	abnormal	1	33,33%	7	20,59%	
BMI < 85	YES	2	66,67%	27	79,41%	0.828
	NO	1	33,33%	7	20,59%	
RR	<90 percentil	3	100,00%	33	97,06%	0.956
	90-95 percentil	0	0,00%	1	2,94%	
	>95 percentil	0	0,00%	0	0,00%	
Ideal cardiovascular health	YES	0	0,00%	0	0,00%	------
	NO	3	100,00%	34	100,00%	
Passive smoking	NO	2	66,67%	34	100,00%	0.806
	YES	1	33,33%	4	11,76%	
Smoking parents	NO	3	100,00%	31	91,18%	0.571
	YES	0	0,00%	3	8,82%	
Family history of cardiovascular disease	NO	2	66,67%	17	50,00%	0.964
	YES	1	33,33%	17	50,00%	
Uric acid	<4mg/L	2	66,67%	17	50,00%	0.964

	>=4mg/L	1	33,33%	17	50,00%	
Fasting glucose	<100 mg/L	3	100,00%	33	97,06%	0.120
	>=100mg/L	0	0,00%	1	2,94%	
HDL	<40 mg/dl	0	0,00%	2	5,88%	0.729
	>45 mg/dl	3	100,00%	28	82,35%	
	40-45 mg/dl	0	0,00%	4	11,76%	
HDL	<40mg/dl	0	0,00%	2	5,88%	0.368
	>45 mg/dl<br>40-45 mg/dl	3	100,00%	32	94,12%	
LDL	<110 mg/dl	0	0,00%	19	55,88%	0.123
	110-129mg/dl	1	33,33%	8	23,53%	
	>130 mg/dl	2	66,67%	7	20,59%	
TC	<170 mg/dl	1	33,33%	21	61,76%	0.478
	170-199 mg/dl	2	66,67%	11	32,35%	
	>200 mg/dl	0	0,00%	2	5,88%	
Triglycerides	0-9 years <75 mg/dl<br>10-19 years <90 mg/dl	1	33,33%	21	61,76%	0.257
	0-9 years 75-99 mg/dl<br>10-19 years 90-129 mg/dl	2	66,67%	8	23,53%	
	0-9 years >100 mg/dl<br>10-19 years >130 mg/dl	0	0,00%	5	14,71%	
Lipid profile	normal	0	0,00%	9	26,47%	0.747
	abnormal	3	100,00%	25	73,53%	
physical activity	No	0	0,00%	7	20,59%	0.916
	any kind of	3	100,00%	27	79,41%	

physical activity	No	0	0,00%	22	64,71%	0.115
	>= 3 days/week	3	100,00%	12	35,29%	
number of days with physical activity >60 min per day	0	0	0,00%	7	20,59%	0.015
	1	0	0,00%	7	20,59%	
	2	0	0,00%	8	23,53%	
	3	1	33,33%	2	5,88%	
	4	0	0,00%	3	8,82%	
	5	0	0,00%	3	8,82%	
	6	1	33,33%	0	0,00%	
	7	1	33,33%	4	11,76%	
Physical activity 7 days a week > 60 min	No	2	66,67%	30	88,24%	0.867
	Yes	1	33,33%	4	11,76%	
Sedetary screen time before Covid pandemy	< 3h	2	66,67%	8	23,53%	0.350
	>= 3h	1	33,33%	26	76,47%	
Sedetary screen time in Covid pandemy	< 3h	0	0,00%	0	0,00%	------
	>= 3h	3	100,00%	34	100,00%	

cIMT—carotid intima-media thickness; BMI—body mass index; ESR—erythrocytes sedimentation rate; CRP—C-reactive protein; RCA—right carotid artery; LCA—left carotid artery. 1—Abnormal BMI encompasses patients with one of the following: underweight (<5th percentile); overweight (>85th percentile); obesity (>95th percentile); 2—Abnormal lipid panel encompasses patients with at least one of the following: total cholesterol ≥ 170 mg/dL; high-density lipoprotein ≤40 mg/dL; low-density lipoprotein ≥ 110 mg/dL; triglycerides 0-9 years >100 mg/dl, 10-19 years >130 mg/dl; AGA- appropriate for gestational age; LGA –large for gestational age; SGA-small for gestational age;
